# Retinal ischemic perivascular lesions in cortical cerebral infarction: a comparison against controls

**DOI:** 10.1038/s41433-026-04680-1

**Published:** 2026-06-27

**Authors:** Jay B. Bisen, Hayden Sikora, Richa Shah, Michael Drakopoulos, Nicholas J. Volpe, Rukhsana G. Mirza

**Affiliations:** Department of Ophthalmology, Northwestern University, Feinberg School of Medicine, Chicago, IL USA

**Keywords:** Predictive markers, Blood flow

## Abstract

**Background/Objectives:**

Retinal ischemic perivascular lesions (RIPLs), identified on optical coherence tomography (OCT) imaging, represent focal retinal infarcts. RIPLs have been linked to stroke, but not specifically cortical ischemic infarctions. Main study questions: Do RIPL counts differ among controls, pre-stroke patients, and post-stroke patients?

**Subjects/Methods:**

Retrospective, cross-sectional, single institution study of 65 identified patients consenting for research who had both high-quality, bilateral, 6 mm × 6 mm macular optical coherence tomography (OCT) imaging performed within five years of cortical ischemic infarction occurrence and 65 controls who had a coronary artery calcium (CAC) score of 0 within 1 year following baseline OCT. The differences in mean total RIPLs among pre-stroke, post-stroke, and controls groups were assessed, as were differences by stroke circulation (anterior vs. posterior) and laterality (ipsilateral vs. contralateral eye). The effects of potential confounders (known cardiovascular risk factors) were examined with multivariate analysis.

**Results:**

129 patients [controls:65; pre-stroke:27; post-stroke:37 (anterior:17; posterior:20)] were included. Pre-stroke and post-stroke patients have significantly higher RIPL counts compared to controls (mean ± std: controls, 2.05 ± 2.41; pre-stroke, 3.39 ± 3.55; post-stroke, 3.62 ± 4.14; *p* = 0.03). No differences were identified between pre- and post-stroke patients, anterior and posterior circulation, and ipsilateral and contralateral eyes. Multivariate analyses identified no statistically significant association between RIPL counts and post-stroke patients compared to controls.

**Conclusions:**

Pre- and post-stroke patients exhibit higher RIPL counts versus controls, without differences between anterior- and posterior-circulation strokes or ipsilateral and contralateral eyes. In cortical stroke, RIPLs likely indicate chronic microvascular dysfunction from baseline cardiovascular risk factors, not acute stroke-associated changes.

## Introduction

Cerebrovascular disease (CVA) is a leading cause of mortality and disability worldwide, making primary prevention essential [1]. Physicians use risk assessment tools such as the American Heart Association (AHA)/American College of Cardiology (ACC) Atherosclerotic Cardiovascular Disease (ASCVD) risk calculator and the Framingham Stroke Profile [2, 3] to guide medical management and reduce CVA risk. However, these tools do not account for acute vascular changes or disease progression, making them less effective for acute assessment and ongoing monitoring [2, 4].

Imaging techniques effectively assess vascular status, with carotid intima-media thickness (cIMT), plaque measurements, and coronary artery calcium (CAC) scores informing CVA risk. However, results can vary among interpreters and require advanced imaging, which may not be widely available [5–8]. CAC scores, specifically, are costly, expose patients to radiation, are not repeatable, and provide limited incremental value to CVA risk prediction [4, 9–11].

Given these limitations, there is a need for sensitive, repeatable, and noninvasive tools to evaluate the central nervous system (CNS) microvascular health for CVA risk assessment. Retinal optical coherence tomography (OCT) offers a rapid and noninvasive assessment of retinal ischemic changes associated with CVA. Notably, retinal artery occlusions (RAO) are considered stroke equivalents [12], and both RAOs and retinal vein occlusions (RVO) significantly increase subsequent stroke risk [13–15]. Given this relationship, the retina’s vulnerability to ischemic injury likely reflects the CNS microvascular health [16].

**Fig. 1:**
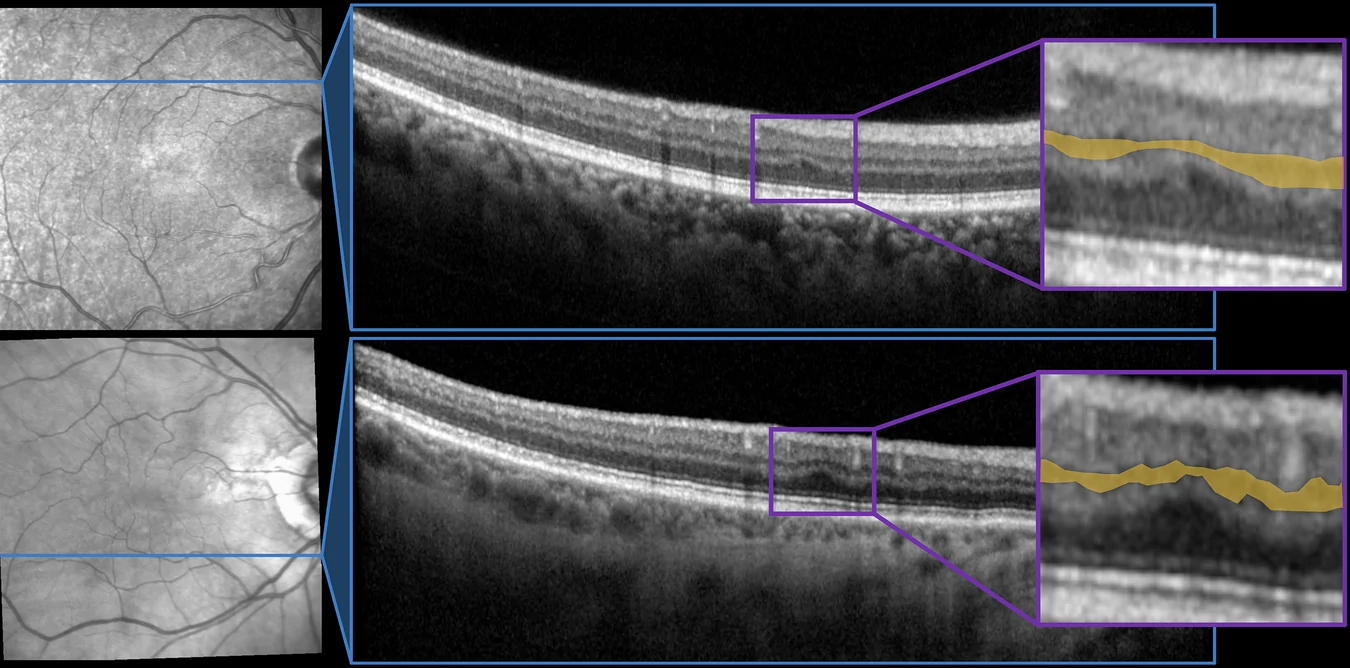
Examples of retinal ischemic perivascular lesions (RIPLs) in two eyes from the pre-stroke group, identified on individual OCT B Scans (Spectralis, EssilorLuxoticca, Paris, France), defined by focal inner nuclear layer (INL) thinning, inward displacement of the outer plexiform layer (OPL), and expansion of the outer nuclear layer (ONL). For each example (top and bottom rows), the corresponding B-scan slice is first localized on near-infrared (NIR) imaging (left panels), with the scan position marked by a horizontal blue line, and then displayed on the OCT B-scan (center panels). The RIPL location is highlighted by a purple box, with a magnified inset (right panels) illustrating the characteristic laminar changes. In the magnified insets, an orange-shaded overlay delineates the segmented INL region, demonstrating focal INL thinning, OPL inward displacement, and compensatory ONL expansion, consistent with RIPL morphology.

**Fig. 2:**
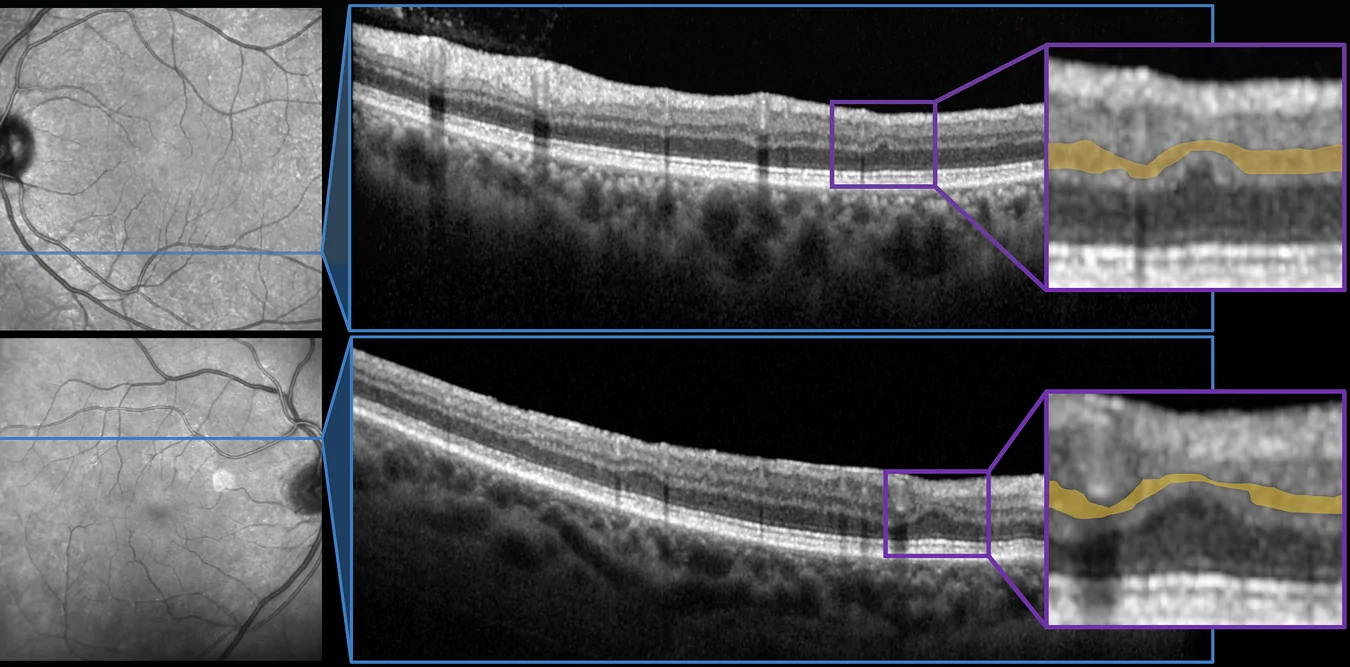
Examples of retinal ischemic perivascular lesions (RIPLs) in two eyes from the post-stroke group, identified on individual OCT B Scans (Spectralis, EssilorLuxoticca, Paris, France), defined by focal inner nuclear layer (INL) thinning, inward displacement of the outer plexiform layer (OPL), and expansion of the outer nuclear layer (ONL). For each example (top and bottom rows), the corresponding B-scan slice is first localized on near-infrared (NIR) imaging (left panels), with the scan position marked by a horizontal blue line, and then displayed on the OCT B-scan (center panels). The RIPL location is highlighted by a purple box, with a magnified inset (right panels) illustrating the characteristic laminar changes. In the magnified insets, an orange-shaded overlay delineates the segmented INL region, demonstrating focal INL thinning, OPL inward displacement, and compensatory ONL expansion, consistent with RIPL morphology.

Retinal ischemic perivascular lesions (RIPL) are a novel retinal OCT biomarker associated with focal infarcts in the middle retina, likely due to hypoperfusion or ischemia in this region [17]. RIPLs are defined as areas of outer nuclear layer (ONL) expansion, outer plexiform layer (OPL) displacement, and inner nuclear layer (INL) thinning [18] (Figs. 1, 2, and Supplementary Fig. 1). These lesions often present asymptomatically in patients with subclinical systemic disease, therefore increasing interest in assessing their association with CVA. Elevated RIPL presence has been associated with subcortical infarctions [19], RVOs [20], and infarctions in atrial fibrillation (AF) [21]. They have also been linked to systemic vascular conditions that carry an increased CVA risk, including hypertension (HTN) [22], carotid artery stenosis (CAS) [23, 24], type-2 diabetes mellitus (T2DM) [25], and AF [26].

The association between RIPLs and CVA suggests they could be a marker of retinal and CNS microvascular dysfunction, but their presence in cortical strokes remains unexplored. This study aims to compare RIPL counts among pre-stroke patients, stroke patients, and controls to better understand whether RIPLs relate more to acute ischemic events or to chronic systemic microvascular damage. Additionally, this study aims to investigate differences in RIPL counts between anterior and posterior circulation strokes, as well as between ipsilateral and contralateral hemispheres, to determine whether regional thromboembolic events in the CNS microcirculation are associated with increased RIPL presence in those regions.

## Materials/subjects and methods

This retrospective, single-institution cross-sectional study included all patients from the Northwestern Memorial Hospital system who underwent retinal OCT imaging with a diagnosis of cortical ischemic stroke within five years of retinal imaging, as determined by the electronic medical record (EMR). This study adhered to the tenets of the Declaration of Helsinki, and Institutional Review Board approval was obtained from Northwestern University in Chicago, Illinois. Patient consent for involvement in retrospective clinical studies was obtained during initial clinical presentation. Patient data was de-identified to ensure privacy and confidentiality.

Patients were excluded if they had a diagnosis of small-vessel cerebral stroke, identified by MRI reports available in the EMR, where cortical strokes were defined as large vessel thromboembolic processes affecting the cerebral cortex, whereas subcortical infarctions were defined as small-vessel infarctions affecting deeper cerebral regions including the basal ganglia, thalamus, or internal capsule. Additionally, patients were excluded if they had significant retinal disease identified through the EMR or retinal imaging, including age-related macular degeneration, diabetic retinopathy, diabetic macular edema, glaucoma, retinal vein occlusion, or retinal artery occlusion. Patients were excluded if they had poor-quality OCT images, defined as those with reduced resolution (blurring or loss of detail impairing reliable retinal layer delineation), motion artifact, shadow artifact, or if imaging was available for only one eye.

Patients with retinal imaging conducted after their stroke were classified as the post-stroke group, while those with imaging before their stroke were designated as pre-stroke patients. Patients with imaging at both timepoints had their imaging included in both respective groups.

The post-stroke group was further subdivided based on both vascular territory (anterior vs. posterior circulation) and hemispheric involvement (ipsilateral vs. contralateral eye). The anterior stroke group included patients with infarctions in the anterior circulation, specifically affecting the anterior cerebral artery (ACA), middle cerebral artery (MCA), and internal carotid artery (ICA). The posterior stroke group included those with infarctions in the posterior circulation, involving the posterior cerebral artery (PCA) and cerebellar arteries. Similarly, ipsilateral and contralateral eye groups were defined according to the hemisphere of the brain affected by the infarction. Patients with strokes affecting both anterior and posterior circulations, or both hemispheres, were excluded from this sub-analysis, allowing for isolated comparisons between these groups.

The control group consisted of patients with a CAC score of 0 measured within one year after OCT imaging. This group was selected because they may have risk factors for CVA, but do not exhibit sequelae of cardiovascular disease or have a history of major adverse cardiovascular events (MACEs), allowing this cohort to serve as an effective comparator for assessing differences between control, pre-stroke, and post-stroke groups. For this group, CAC scoring was performed using the Agatston method [27], with scores extracted directly from the EMR.

Retinal imaging consisted of 6 mm × 6 mm 768-pixel-wide macular OCT scans (Spectralis, EssilorLuxoticca, Paris, France) with 61 B-scans each. RIPLs were defined as focal regions of inner nuclear layer thinning, outer plexiform layer inward deviation, and outer nuclear layer expansion. OCT images were assessed for RIPL presence independently by two graders (JB, HS), with discrepancies resolved by an arbitrator (MD), all blinded to stroke status. The arbitrator was used in 35% of all identified RIPLs. All graders had prior experience with RIPL grading on SD-OCT. RIPLs seen on multiple consecutive OCT B-scans were counted only once. RIPL counts were the summed RIPL counts between both eyes except when comparing ipsilateral and contralateral eyes.

Clinical and demographic data, including age, sex, HTN, T2DM, smoking status, chronic kidney disease (CKD), peripheral arterial disease (PAD), AF, CAS, CVD history, hypercholesterolemia, and cholesterol levels (closest blood draw to retinal OCT imaging), were extracted from the EMR. Patients were considered positive for CVD history if they had coronary artery disease, valvular disease (including aortic stenosis and mitral valve prolapse), cardiomyopathies, arrhythmias (including atrial flutter, atrial fibrillation, and paroxysmal supraventricular tachycardia), aortic disease, or pulmonary vasculature disorders (including pulmonary arterial hypertension). Comorbidities were determined based on documented medical history and diagnoses within the EMR.

Clinical and demographic characteristics and RIPL counts were compared among controls, pre stroke, and post stroke groups and between anterior and posterior stroke and ipsilateral and contralateral hemispheric groups. Categorical variables, such as the percentage of patients with HTN, T2DM, CKD, PAD, AF, CAS, active smoking status, CVD history, and hypercholesterolemia, were compared using Pearson Chi-squared tests. Continuous variables such as LDL, HDL, triglyceride, total cholesterol levels and RIPL counts were compared using Kruskal–Wallis testing. Post-hoc testing, Fisher-Exact testing, and Dunn’s testing with Bonferroni correction, respectively, were performed to assess differences between each group individually.

Previous studies have identified a significant increase in the odds of patients having CVD with RIPL counts greater than one [28]. Therefore, total RIPL counts were also stratified into categorial groups [Low RIPL Count: 0–1; High RIPL Count: 2+] and analyzed using Pearson Chi-squared testing, with subsequent post-hoc Fisher-Exact testing.

Univariate and binary multivariate logistic regression models were used to assess whether total RIPL burden, categorized as low (0–1) or high (≥2), was independently associated with stroke status, post-stroke versus control. Covariates included sex, HTN, CAS, CVD history, and BMI. Variance inflation factors were checked for collinearity. Adjusted odds ratios, 95% confidence intervals, and two-sided *p*-values are reported, with *p*  <  0.05 considered statistically significant.

## Results

129 patients were included in the study after exclusions (Supplementary Fig. 2), with 65 control patients (CAC = 0), 27 pre-stroke patients (retinal imaging before stroke occurrence), and 37 post-stroke patients (retinal imaging after stroke occurrence). Of the 37 post-stroke patients, 20 patients had posterior strokes, while 17 had anterior strokes. 34 post-stroke patients had unilateral strokes, and all 68 eyes were separated into ipsilateral and contralateral groups. Five of the 37 post-stroke patients had retinal imaging before stroke incidence. Notably, retinal OCT images in the pre-stroke group were obtained an average of 1.30 years before stroke occurrence, while those in the post-stroke group were taken on average 2.09 years after stroke occurrence.

Demographic and clinical characteristic analyses identified a statistically significant difference in sex, CKD, AF, CAS, and CVD history incidence when comparing all three groups (Table 1). There was also a significant difference in the rates of antihypertensive, lipid lowering, and anticoagulant/antiplatelet (ACAP) treatment (Table 1). Additional comparisons are reported in Supplementary Table 1.

**Table 1:** Significant demographic and clinical characteristics of the overall cohort, with a comparison between the control (C), pre-stroke (PeS), and post-stroke (PoS) groups.

Characteristic	Overall *N* = 129^a^	Control *N* = 65^a^	Pre-stroke *N* = 27^a^	Post-stroke *N* = 37^a^	C vs. PeS vs. PoS *P*-value	C vs. PeS *P*-value	C vs. PoS *P*-value	PeS vs. PoS *P*-value
Sex^b,c^					<0.001*	0.06	<0.001*	0.21
Male	41 (32%)	11 (17%)	10 (36%)	20 (54%)				
Female	89 (68%)	54 (83%)	18 (64%)	17 (46%)				
Hypertension^b,c^					0.002*	0.001*	0.13	0.13
No	75 (58%)	46 (71%)	9 (32%)	20 (54%)				
Yes	55 (42%)	19 (29%)	19 (68%)	17 (46%)				
Chronic kidney disease^b,c^					<0.001*	<0.001*	<0.001*	0.77
No	114 (88%)	65 (100%)	22 (79%)	27 (73%)				
Yes	16 (12%)	0 (0%)	6 (21%)	10 (27%)				
Atrial fibrillation^b,c^					0.002*	0.03*	<0.001*	0.49
No	120 (92%)	65 (100%)	25 (89%)	30 (81%)				
Yes	10 (8%)	0 (0%)	3 (11%)	7 (19%)				
Carotid artery stenosis^b,c^					0.28	<0.001*	<0.001*	1.00
No	107 (82%)	62 (95%)	19 (68%)	26 (70%)				
Yes	23 (18%)	3 (5%)	9 (32%)	11 (30%)				
History of CVD^b,c^					<0.001*	<0.001*	<0.001*	0.31
No	80 (62%)	55 (85%)	13 (46%)	12 (44%)				
Yes	50 (38%)	10 (15%)	15 (54%)	25 (56%)				
Anti-hypertensive medications^b,c^					<0.001*	<0.001*	0.001*	0.17
No	63 (48%)	45 (69%)	5 (18%)	13 (35%)				
Yes	67 (52%)	20 (31%)	23 (82%)	24 (65%)				
Anticoagulant and antiplatelet medications^b,c^					<0.001*	<0.001*	<0.001*	0.28
No	66 (51%)	58 (89%)	5 (18%)	3 (8%)				
Yes	64 (49%)	7 (11%)	23 (82%)	34 (92%)				
Lipid lowering medications^b,c^					<0.001*	<0.001*	<0.001*	0.58
No	65 (50%)	47 (72%)	9 (32%)	9 (24%)				
Yes	65 (50%)	18 (28%)	19 (68%)	28 (76%)				

^a^Mean ± (SD); *n* (%).

^b^Fisher’s exact test.

^c^Pearson’s Chi-squared test.

The bold and astericks indicate statistical significance.

When individually assessed against controls using post-hoc testing, pre- and post-stroke patients had significantly higher incidence of CKD, AF, CAS, and CVD history compared to controls (Table 1). Those patients also had significantly increased antihypertensive, ACAP, and statin use compared to controls (Table 1). Pre-stroke patients had a significantly higher incidence of HTN, and post-stroke patients had a significant male predominance when compared to controls. No significant differences between demographic and clinical characteristics were observed between the pre- and post-stroke (Table 1; Supplementary Table 1) and anterior and posterior circulation stroke groups (Supplementary Table 2).

When comparing the controls, pre-stroke, and post-stroke patients, there was a significant difference between RIPL counts (Means: Control: 2.05; Pre: 3.39; Post: 3.62; *p* = 0.03; Table 2) between the three groups. Post hoc analyses identified that post-stroke patients had significantly higher RIPL counts (*p* = 0.04; Table 2), and both pre- (Means: Control: 48%; Pre: 74%; *p* = 0.02; Table 2) and post-stroke (Means: Control: 48%; Post: 70%; *p* = 0.04; Table 2) patients had significantly higher elevated RIPL presence (RIPLs ≥ 2), when compared to controls. Notably, no significant differences between RIPL presence were observed between the anterior and posterior circulation stroke groups and ipsilateral and contralateral groups (Supplementary Tables 3, 4).

**Table 2:** Comparison of retinal ischemic perivascular lesions between the control (C), pre-stroke (PeS), and post-stroke (PoS) groups.

Characteristic	Overall *N* = 129^a^	Control *N* = 65^a^	Pre-Stroke *N* = 27^a^	Post-Stroke *N* = 37^a^	C vs. PeS vs. PoS *P* value	C vs. PeS *P*-value	C vs. PoS *P*-value	PeS vs. PoS *P*-value
RIPL score^b,c^	2.79 ± 3.29	2.05 ± 2.41	3.39 ± 3.55	3.62 ± 4.14	0.03*	0.27	0.04*	1.00
Categorized RIPL score^d,e^					0.06	0.02*	0.04*	0.79
0-1	55 (42%)	34 (52%)	10 (26%)	11 (30%)				
2+	75 (58%)	31 (48%)	18 (74%)	26 (70%)				

^a^Mean ± (SD); *n* (%).

^b^Dunn’s test with Bonferroni Correction.

^c^Kruskal–Wallis test.

^d^Fisher’s exact test.

^e^Pearson’s Chi-squared test.

The bold and astericks indicate statistical significance.

Among all observations (*N* = 129), 56 participants (43.4%) had bilateral RIPLs, 43 (33.3%) had unilateral involvement (OD only: 17, 13.2%; OS only: 26, 20.2%), and 30 (23.3%) had no RIPLs in either eye. Bilateral involvement was most common in the pre-stroke (14/27, 51.9%) and post-stroke (19/37, 51.4%) groups, compared to controls (23/65, 35.4%). Of participants with any RIPLs (*N* = 99), bilaterality was present in 56 (56.6%), including 23/46 (50.0%) controls, 19/32 (59.4%) post-stroke, and 14/21 (66.7%) pre-stroke participants (Supplementary Table 5).

Univariate logistic regression identified that post-stroke patients exhibited higher odds of elevated RIPL presence (≥2 RIPLs) (OR: 2.59, 95% CI: 1.10–6.11, *p* = 0.03; Supplementary Table 6) compared to controls.

After multivariate correction, there was no statistically significant increased odds of elevated RIPL presence (OR: 1.12, 95% CI: 0.92–1.37, *p* = 0.24; Table 4) in the post-stroke group. However, post-stroke status was significantly associated with higher odds of CVD history and male sex (Table 3).

**Table 3:** Multivariate logistic regression models assessing the relationship between elevated retinal ischemic perivascular lesions (RIPL) presence (RIPL ≥ 2) and decreased RIPL (RIPL < 2) presence between the control and post-stroke group.

Characteristic	Variance inflation factor	Odds ratio	95% confidence interval	Beta coefficient	*P*-value
Population					
Control	–	–	–	–	
Post-stroke	1.09	1.76	0.59–5.22	0.567	0.31
Sex					
Female	–	–	–	–	
Male	1.20	3.61	1.17–11.24	1.286	0.03*
Hypertension					
No	–	–	–	–	
Yes	1.09	1.56	0.51–4.82	0.446	0.44
Carotid artery stenosis					
No	–	–	–	–	
Yes	1.25	3.79	0.64–22.28	1.332	0.14
CVD history					
No	–	–	–	–	
Yes	1.37	5.60	1.86–16.84	1.723	0.002*
BMI	1.13	1.04	0.97–1.11	0.039	0.26

The bold and astericks indicate statistical significance.

## Discussion

This study identifies a significant increase in RIPL burden in patients before and after stroke occurrence relative to controls. In addition to an increased RIPL burden, pre- and post-stroke patients had a significantly greater prevalence of comorbid risk factors. Interestingly, our statistical comparisons recognized no significant difference between patients before and after stroke incidence, anterior and posterior circulation strokes, and differences in hemispheric presentation. After adjusting for significant clinical covariates, some of which directly contribute to RIPL development, such as HTN [22], CAS [23, 24], and CVD history [28], the association between RIPLs and post-stroke status was no longer significant.

Our findings do not show an independent relationship between RIPLs and cortical ischemic stroke. Instead, RIPL formation in these patients appears associated with chronic systemic vascular disease. We suggest that RIPLs may serve as markers of chronic microvascular dysfunction, reflecting systemic vascular disease rather than acute thromboembolic events. This supports the view that RIPLs signal chronic vascular pathology and increased risk for MACEs, such as myocardial infarction [29] and cerebral infarction [19, 21].

These findings suggest that the burden of retinal OCT-detected RIPLs could serve as a practical marker of cumulative microvascular and cardiometabolic disease. This is particularly relevant for neurologists caring for patients with stroke or elevated vascular risk. By documenting RIPLs, clinicians may better identify individuals who require intensified secondary prevention, closer monitoring, and targeted evaluation for systemic vascular contributors such as carotid stenosis or atrial fibrillation. If validated in future studies, incorporating RIPL assessment into multidisciplinary stroke clinics could offer a rapid, noninvasive ocular biomarker to assess CNS microvascular status and support macrovascular risk stratification in conjunction with neuroimaging and established risk metrics.

Our results build on prior research showing higher RIPL prevalence in patients with acute cerebrovascular events, including subcortical strokes [19], CVA in AF [21], and RVO [20]. However, in contrast to those studies, we did not find an independent association between RIPL counts and stroke after adjusting for covariates. This difference may be due to the distinct mechanisms underlying cortical strokes, often resulting from embolic or atherosclerotic large vessel disease, in comparison to subcortical strokes and RVO [30], which are related to hypertensive or T2DM-related small-vessel disease [30].

RIPLs are focal areas of middle retinal infarction caused by hypoperfusion of the deep capillary plexus (DCP), typically in a perivenular pattern where oxygen tension is lowest. INL thinning indicates chronic, focal ischemic injury [31–33]. In contrast, OPL displacement and ONL expansion are likely secondary changes from middle retinal injury, driven by altered neuroglial support, microvascular permeability, and local tissue reorganization, rather than primary outer retinal infarction [32]. Eyes with RIPLs show reduced deep vascular complex density on OCT angiography, supporting focal capillary dropout and chronic DCP compromise [34]. RIPLs are part of a broader “retinal ischemic cascade” that begins with perivenular PAMM lesions and progresses with increasing ischemia [33]. Retinal ischemia causes metabolic stress and mitochondrial dysfunction in neurons, along with immune activation, proinflammatory signaling, and endothelial injury, which worsen tissue damage and disrupt barriers [35–37]. Müller glia are key for retinal metabolism and barrier integrity; ischemia-related inflammation can impair glial function, leading to laminar disorganization and secondary OPL and ONL changes near INL infarcts [35–37].

Given their pathophysiologic development, RIPLs, in the context of cortical strokes, may indicate underlying chronic vascular health rather than acute stroke etiology. Despite the lack of an independent association, RIPLs remain valuable as markers of vascular dysfunction and are more frequently observed in patients with CVA and CVD. This relationship between RIPLs and stroke-related pathology aligns with broader evidence linking retinal vascular abnormalities to systemic cardiovascular disease, underscoring the value of retinal OCT imaging as a sensitive tool in vascular risk assessment. Beyond cerebrovascular disease, RIPLs have been associated with a range of cardiovascular conditions, including hypertension [22], carotid artery stenosis [23, 24], atrial fibrillation [26], and myocardial infarction [29].

Additionally, our analysis found notable differences in medication use between groups, likely reflecting the initiation of antihypertensive and lipid-lowering therapies prior to stroke and the introduction of ACAP after the ischemic event. Despite medical management, both pre- and post-stroke patients continued to show elevated BMI, decreased HDL, and increased triglycerides, highlighting persistent metabolic risk factors that elevate ischemic event risk. Unexpectedly, HbA1c levels, T2DM prevalence, and smoking status were similar across groups, possibly due to the control group selection. Controls likely had underlying diseases warranting CAC testing and retinal imaging, as suggested by their average RIPL counts, which were near two, a threshold linked with higher CVD risk [28]. Given the high prevalence of T2DM and HTN in our study population, the lack of significant differences in these variables across groups is intriguing but not unexpected.

Several limitations should be considered when contextualizing our findings. Given the cross-sectional design, the smaller sample size, and the characteristics of the control group, further longitudinal studies with larger, well-matched cohorts are needed to elucidate the etiological significance of RIPLs and to determine any causal relationship with cortical ischemic stroke. Additionally, post-stroke cognitive impairment remains a major concern, with evidence suggesting that post-stroke microvascular dysfunction plays a significant role in cognitive decline [38–41]. Given the growing evidence that RIPLs reflect systemic vascular dysfunction, further research should explore their relationship with stroke severity and long-term cognitive recovery.

These findings suggest that RIPLs may be surrogate markers of systemic microvascular health. Noninvasive retinal OCT imaging allows visualization of the CNS and its microvascular characteristics, potentially providing clinicians with a means to assess and characterize vascular changes that were previously inaccessible. RIPLs might indicate early underlying vascular dysfunction and, in the context of cortical cerebral infarctions, could offer insights into cerebrovascular health and possible infarction risk.

### What was known before:

- RIPLs have been associated with cardiovascular disease. The association of RIPLs with cortical cerebral infarction is unknown.
- RIPLs might reflect general cardiovascular risk, including risk of cortical cerebral infarction.

### What this study adds:

- RIPLs are associated with cortical cerebral infarction. However, this risk is not independent of known cardiovascular disease risk factors.
- RIPLs might identify individuals with cardiovascular disease.

## Supplementary information

## Data Availability

Deidentified data from this study will be available upon reasonable request made to the corresponding author (r-mirza@northwestern.edu) for 6 months to 3 years after publication, pending author Institutional Review Board approval and requestor execution of a data‑use agreement with Northwestern.

## References

[1] Pu L, Wang L, Zhang R, Zhao T, Jiang Y, Han L. Projected global trends in ischemic stroke incidence, deaths and disability-adjusted life years from 2020 to 2030. Stroke. 2023;54:1330–9. 37094034 10.1161/STROKEAHA.122.040073

[2] Meschia JF, Bushnell C, Boden-Albala B, Braun LT, Bravata DM, Chaturvedi S, et al. Guidelines for the primary prevention of stroke: a statement for healthcare professionals from the American Heart Association/American Stroke Association. Stroke. 2014;45:3754–832. 25355838 10.1161/STR.0000000000000046 PMC5020564

[3] Ruksakulpiwat S. Stroke Risk Screening Scales (SRSS): identification of domain and item generation. J Stroke Cerebrovasc Dis. 2021;30:105740. 33761449 10.1016/j.jstrokecerebrovasdis.2021.105740

[4] Osawa K, Nakanishi R, McClelland RL, Polak JF, Bishop W, Sacco RL, et al. Ischemic stroke/transient ischemic attack events and carotid artery disease in the absence of or with minimal coronary artery calcification: results from the Multi-Ethnic Study of Atherosclerosis. Atherosclerosis. 2018;275:22–7. 29852401 10.1016/j.atherosclerosis.2018.05.027 PMC6113099

[5] Lorenz MW, Markus HS, Bots ML, Rosvall M, Sitzer M. Prediction of clinical cardiovascular events with carotid intima-media thickness: a systematic review and meta-analysis. Circulation. 2007;115:459–67. 17242284 10.1161/CIRCULATIONAHA.106.628875

[6] Den Ruijter HM, Peters SA, Anderson TJ, Britton AR, Dekker JM, Eijkemans MJ, et al. Common carotid intima-media thickness measurements in cardiovascular risk prediction: a meta-analysis. JAMA. 2012;308:796–803. 22910757 10.1001/jama.2012.9630

[7] Naqvi TZ, Lee MS. Carotid intima-media thickness and plaque in cardiovascular risk assessment. JACC Cardiovasc Imaging. 2014;7:1025–38. 25051948 10.1016/j.jcmg.2013.11.014

[8] Saba L, Antignani PL, Gupta A, Cau R, Paraskevas KI, Poredos P, et al. International Union of Angiology (IUA) consensus paper on imaging strategies in atherosclerotic carotid artery imaging: from basic strategies to advanced approaches. Atherosclerosis. 2022;354:23–40. 35816927 10.1016/j.atherosclerosis.2022.06.1014

[9] Cortiana V, Vaghela H, Bakhle R, Santhosh T, Kaiwan O, Tausif A, et al. Beyond the heart: the predictive role of coronary artery calcium scoring in non-cardiovascular disease risk stratification. Diagnostics. 2024;14:2349. 10.3390/diagnostics14212349 PMC11545064 39518317

[10] Kwok CS, Bennett S, Lip GYH. Coronary artery calcium score and its association with stroke: a systematic review and meta-analysis. Eur J Clin Invest. 2023;53:e13892. 36251530 10.1111/eci.13892

[11] Baber U, Mehran R, Sartori S, Schoos MM, Sillesen H, Muntendam P, et al. Prevalence, impact, and predictive value of detecting subclinical coronary and carotid atherosclerosis in asymptomatic adults: the BioImage study. J Am Coll Cardiol. 2015;65:1065–74. 25790876 10.1016/j.jacc.2015.01.017

[12] Mac Grory B, Schrag M, Biousse V, Furie KL, Gerhard-Herman M, Lavin PJ, et al. Management of central retinal artery occlusion: a scientific statement from the American Heart Association. Stroke. 2021;52:e282–e94. 33677974 10.1161/STR.0000000000000366

[13] Lavin P, Patrylo M, Hollar M, Espaillat KB, Kirshner H, Schrag M. Stroke risk and risk factors in patients with central retinal artery occlusion. Am J Ophthalmol. 2019;200:271–2. 30827486 10.1016/j.ajo.2019.01.021

[14] Bakhoum CY, Madala S, Long CK, Adabifirouzjaei F, Freeman WR, Goldbaum MH, et al. Retinal vein occlusion is associated with stroke independent of underlying cardiovascular disease. Eye. 2023;37:764–7. 35411111 10.1038/s41433-022-02038-x PMC9998396

[15] Wu CY, Riangwiwat T, Limpruttidham N, Rattanawong P, Rosen RB, Deobhakta A. Association of retinal vein occlusion with cardiovascular events and mortality: a systematic review and meta-analysis. Retina. 2019;39:1635–45. 30829987 10.1097/IAE.0000000000002472

[16] Colcombe J, Mundae R, Kaiser A, Bijon J, Modi Y. Retinal findings and cardiovascular risk: prognostic conditions, novel biomarkers, and emerging image analysis techniques. J Pers Med. 2023;13:1564. 10.3390/jpm13111564 PMC10672409 38003879

[17] Limoli C, Khalid H, Wagner SK, Huemer J. Retinal ischemic perivascular lesions (RIPLs) as potential biomarkers for systemic vascular diseases: a narrative review of the literature. Ophthalmol Ther. 2025;14:1183–97. 40293678 10.1007/s40123-025-01148-5 PMC12069769

[18] Madala S, Adabifirouzjaei F, Lando L, Yarmohammadi A, Long CP, Bakhoum CY, et al. Retinal ischemic perivascular lesions, a biomarker of cardiovascular disease. Ophthalmol Retin. 2022;6:865–7. 10.1016/j.oret.2022.05.005 PMC9464692 35589077

[19] Kwapong WR, Yan Y, Cao L, Wang H, Ye C, Jiang S, et al. Retinal ischemic perivascular lesion reflects cerebral small vessel disease burden in single subcortical infarction. J Am Heart Assoc. 2024;13:e033081. 38639343 10.1161/JAHA.123.033081 PMC11179934

[20] Maltsev DS, Kulikov AN, Burnasheva MA, Chhablani J. Prevalence of resolved paracentral acute middle maculopathy lesions in fellow eyes of patients with unilateral retinal vein occlusion. Acta Ophthalmol. 2020;98:e22–e8. 31347293 10.1111/aos.14196

[21] Bakhoum CY, Au A, Bousquet E, Matesva M, Singer MB, Jayaraj C, et al. Retinal ischemic perivascular lesions are associated with stroke in individuals with atrial fibrillation. J Am Heart Assoc. 2024;13:e035079. 39190603 10.1161/JAHA.123.035079 PMC11646541

[22] Burnasheva MA, Maltsev DS, Kulikov AN, Sherbakova KA, Barsukov AV. Association of chronic paracentral acute middle maculopathy lesions with hypertension. Ophthalmol Retin. 2020;4:504–9. 10.1016/j.oret.2019.12.001 31948908

[23] Drakopoulos M, Zhang DL, Cheng BT, Sadiq SA, Nadel A, Marchese A, et al. Swept-source optical coherence tomography angiography metrics of retinal ischaemic perivascular lesions in patients being evaluated for carotid artery stenosis and controls. BMJ Open Ophthalmol. 2023;8:e001226. 10.1136/bmjophth-2022-001226 PMC10357701 37493668

[24] Zhang DL, Zhang KX, Cheng BT, Heisel CJ, Nadel A, Eskandari MK, et al. Retinal ischemic perivascular lesions are increased in carotid artery stenosis. Ophthalmol Retin. 2023;7:1020–2. 10.1016/j.oret.2023.07.018 37495017

[25] Maltsev DS, Kulikov AN, Burnasheva MA. Association of resolved paracentral acute middle maculopathy lesions with diabetic retinopathy. J Curr Ophthalmol. 2022;34:318–22. 36644464 10.4103/joco.joco_91_22 PMC9832451

[26] Bakhoum CY, Madala S, Lando L, Yarmohammadi A, Long CP, Miguez S, et al. Retinal ischemic perivascular lesions in individuals with atrial fibrillation. J Am Heart Assoc. 2023;12:e028853. 37577936 10.1161/JAHA.122.028853 PMC10492933

[27] Berman DS, Arnson Y, Rozanski A. Coronary artery calcium scanning: the Agatston score and beyond. JACC Cardiovasc Imaging. 2016;9:1417–9. 27931526 10.1016/j.jcmg.2016.05.020

[28] Long CP, Chan AX, Bakhoum CY, Toomey CB, Madala S, Garg AK, et al. Prevalence of subclinical retinal ischemia in patients with cardiovascular disease - a hypothesis driven study. EClinicalMedicine. 2021;33:100775. 33842865 10.1016/j.eclinm.2021.100775 PMC8020165

[29] Bousquet E, Santina A, Au A, Somisetty S, Abraham N, Voichanski S, et al. Retinal ischemic perivascular lesions are associated with myocardial infarction in patients with coronary artery disease. Am J Ophthalmol. 2024;264:224–8. 38552932 10.1016/j.ajo.2024.03.017

[30] Frost E, Kamen S, Oak S, Higham C, Thau L, Vigilante N, et al. Clinical and radiographic phenotypes of patients with multifocal subcortical versus cortical cerebral infarcts. J Stroke Cerebrovasc Dis. 2022;31:106750. 36084434 10.1016/j.jstrokecerebrovasdis.2022.106750

[31] Scharf J, Freund KB, Sadda S, Sarraf D. Paracentral acute middle maculopathy and the organization of the retinal capillary plexuses. Prog Retin Eye Res. 2021;81:100884. 32783959 10.1016/j.preteyeres.2020.100884

[32] Sikora HF, Bisen JB, Heisel CJ, Drakopoulos M, Lyon AT, Mirza RG. Acute precursor of retinal ischemic perivascular lesion identified on optical coherence tomography b-scans in two patients with different etiologies. Retinal Cases Brief Rep. 9900:10.1097/ICB.0000000000001835. 41213000

[33] Abtahi SH, Nourinia R, Mazloumi M, Nouri H, Arevalo JF, Ahmadieh H. Retinal ischemic cascade: new insights into the pathophysiology and imaging findings. Surv Ophthalmol. 2023;68:380–7. 36464134 10.1016/j.survophthal.2022.11.009

[34] Drakopoulos M, Zhang DL, Cheng B, Heisel C, Zhang K, Nadel A, et al. Small retinal ischemic perivascular lesions display quantitative changes on swept source optical coherence tomography angiography. Invest Ophthalmol Vis Sci. 2023;64:PB0053–PB.

[35] Chen Y, Xia Q, Zeng Y, Zhang Y, Zhang M. Regulations of retinal inflammation: focusing on Müller Glia. Front Cell Dev Biol. 2022;10:898652. 35573676 10.3389/fcell.2022.898652 PMC9091449

[36] Ruan Y, Jiang S, Musayeva A, Gericke A. Oxidative stress and vascular dysfunction in the retina: therapeutic strategies. Antioxidants. 2020;9:761. 32824523 10.3390/antiox9080761 PMC7465265

[37] Srejovic JV, Muric MD, Jakovljevic VL, Srejovic IM, Sreckovic SB, Petrovic NT, et al. Molecular and Cellular Mechanisms Involved in the Pathophysiology of Retinal Vascular Disease-Interplay Between Inflammation and Oxidative Stress. Int J Mol Sci. 2024;25:11850. 10.3390/ijms252111850 PMC11546760 39519401

[38] Kalaria RN, Akinyemi R, Ihara M. Stroke injury, cognitive impairment and vascular dementia. Biochim Biophys Acta. 2016;1862:915–25. 26806700 10.1016/j.bbadis.2016.01.015 PMC4827373

[39] Zhang CE, Wong SM, Uiterwijk R, Staals J, Backes WH, Hoff EI, et al. Intravoxel incoherent motion imaging in small vessel disease: microstructural integrity and microvascular perfusion related to cognition. Stroke. 2017;48:658–63. 28196940 10.1161/STROKEAHA.116.015084

[40] Sagnier S, Okubo G, Catheline G, Munsch F, Bigourdan A, Debruxelles S, et al. Chronic cortical cerebral microinfarcts slow down cognitive recovery after acute ischemic stroke. Stroke. 2019;50:1430–6. 31084336 10.1161/STROKEAHA.118.024672

[41] Rensma SP, van Sloten TT, Houben A, Köhler S, van Boxtel MPJ, Berendschot T, et al. Microvascular dysfunction is associated with worse cognitive performance: the Maastricht study. Hypertension. 2020;75:237–45. 31735081 10.1161/HYPERTENSIONAHA.119.13023

