# Supplementary material for: Retinal ischemic perivascular lesions in cortical cerebral infarction: a comparison against controls
Source: Eye (Lond). 2026 Jun 27;40(13):2100–6. doi: 10.1038/s41433-026-04680-1 (PMC13538709; doi:10.1038/s41433-026-04680-1)
Supplement: Supplementary file 1 [file 41433_2026_4680_MOESM1_ESM.docx]

**Supplementary Figure 1: Examples of retinal ischemic perivascular lesions (RIPLs) in two eyes from the control group, identified on individual OCT B Scans (Spectralis, EssilorLuxoticca, Paris, France), defined by focal inner nuclear layer (INL) thinning, inward displacement of the outer plexiform layer (OPL), and expansion of the outer nuclear layer (ONL).** For each example (top and bottom rows), the corresponding B-scan slice is first localized on near-infrared (NIR) imaging (left panels), with the scan position marked by a horizontal blue line, and then displayed on the OCT B-scan (center panels). The RIPL location is highlighted by a purple box, with a magnified inset (right panels) illustrating the characteristic laminar changes. In the magnified insets, a orange-shaded overlay delineates the segmented INL region, demonstrating focal INL thinning, OPL inward displacement, and compensatory ONL expansion, consistent with RIPL morphology.
