# Supplementary material for: Retinal ischemic perivascular lesions in cortical cerebral infarction: a comparison against controls
Source: Eye (Lond). 2026 Jun 27;40(13):2100–6. doi: 10.1038/s41433-026-04680-1 (PMC13538709; doi:10.1038/s41433-026-04680-1)
Supplement: Supplementary file 2 [file 41433_2026_4680_MOESM2_ESM.docx]

**Supplementary Figure 2:** Flow chart detailing stepwise exclusions to determine the final cohort.
