# Supplementary material for: Retinal ischemic perivascular lesions in cortical cerebral infarction: a comparison against controls
Source: Eye (Lond). 2026 Jun 27;40(13):2100–6. doi: 10.1038/s41433-026-04680-1 (PMC13538709; doi:10.1038/s41433-026-04680-1)
Supplement: Supplementary file 3 [file 41433_2026_4680_MOESM3_ESM.docx]

**Supplementary Table 1:** All Demographic and clinical characteristics of the overall cohort, with a comparison between the control (C), pre-stroke (PeS), and post-stroke (PoS) groups

| **Characteristic** | **Overall**  **N = 129** ^1^ | **Control**  **N = 65** ^1^ | **Pre-Stroke**  **N = 27** ^1^ | **Post-Stroke**  **N = 37** ^1^ | **C vs. PeS vs. PoS**  **P-value** | **C vs. PeS**  **P-value** | **C vs. PoS**  **P-value** | **PeS vs. PeS**  **P-value** |
| --- | --- | --- | --- | --- | --- | --- | --- | --- |
| **Age** ^2,3^ | 62.1 ± 12.8 | 62.6 ± 9.7 | 61.0 ± 14.8 | 61.9 ± 15.9 | 0.94 | 1.00 | 1.00 | 1.00 |
| **Sex** ^4,5^ |  |  |  |  | **<0.001*** | 0.06 | **<0.001*** | 0.21 |
| Male | 41 (32%) | 11 (17%) | 10 (36%) | 20 (54%) |  |  |  |  |
| Female | 89 (68%) | 54 (83%) | 18 (64%) | 17 (46%) |  |  |  |  |
| **Hypertension** ^4,5^ |  |  |  |  | **0.002*** | **0.001*** | 0.13 | 0.13 |
| No | 75 (58%) | 46 (71%) | 9 (32%) | 20 (54%) |  |  |  |  |
| Yes | 55 (42%) | 19 (29%) | 19 (68%) | 17 (46%) |  |  |  |  |
| **Type 2 Diabetes Mellitus** ^4,5^ |  |  |  |  | 0.10 | 0.45 | 0.11 | 0.74 |
| No | 114 (88%) | 60 (92%) | 24 (86%) | 30 (81%) |  |  |  |  |
| Yes | 16 (12%) | 5 (8%) | 4 (14%) | 7 (19%) |  |  |  |  |
| **HbA1c** ^2,3^ | 5.68 ± 0.69 | 5.60 ± 0.57 | 5.87 ± 0.91 | 5.66 ± 0.61 | 0.23 | 0.28 | 0.98 | 1.00 |
| **Smoking Status** ^5^ |  |  |  |  | 0.99 | 0.99 | 0.94 | 0.93 |
| Never | 87 (67%) | 44 (68%) | 19 (68%) | 24 (65%) |  |  |  |  |
| Former | 39 (30%) | 19 (29%) | 8 (29%) | 12 (32%) |  |  |  |  |
| Current | 4 (3%) | 2 (3%) | 1 (3%) | 1 (3%) |  |  |  |  |
| **Chronic Kidney Disease** ^4,5^ |  |  |  |  | **<0.001*** | **<0.001*** | **<0.001*** | 0.77 |
| No | 114 (88%) | 65 (100%) | 22 (79%) | 27 (73%) |  |  |  |  |
| Yes | 16 (12%) | 0 (0%) | 6 (21%) | 10 (27%) |  |  |  |  |
| **Peripheral Artery Disease** ^4,5^ |  |  |  |  | 1.00 | 1.00 | 1.00 | 1.00 |
| No | 130 (0%) | 65 (100%) | 28 (100%) | 37 (100%) |  |  |  |  |
| Yes | 0 (0%) | 0 (0%) | 0 (0%) | 0 (0%) |  |  |  |  |
| **Atrial Fibrillation** ^4,5^ |  |  |  |  | **0.002*** | **0.03*** | **<0.001*** | 0.49 |
| No | 120 (92%) | 65 (100%) | 25 (89%) | 30 (81%) |  |  |  |  |
| Yes | 10 (8%) | 0 (0%) | 3 (11%) | 7 (19%) |  |  |  |  |
| **Carotid Artery Stenosis** ^4,5^ |  |  |  |  | 0.28 | **<0.001*** | **<0.001*** | 1.00 |
| No | 107 (82%) | 62 (95%) | 19 (68%) | 26 (70%) |  |  |  |  |
| Yes | 23 (18%) | 3 (5%) | 9 (32%) | 11 (30%) |  |  |  |  |
| **History of CVD** ^4,5^ |  |  |  |  | **<0.001*** | **<0.001*** | **<0.001*** | 0.31 |
| No | 80 (62%) | 55 (85%) | 13 (46%) | 12 (44%) |  |  |  |  |
| Yes | 50 (38%) | 10 (15%) | 15 (54%) | 25 (56%) |  |  |  |  |
| **Anti-hypertensive Medications** ^4,5^ |  |  |  |  | **<0.001*** | **<0.001*** | **0.001*** | 0.17 |
| No | 63 (48%) | 45 (69%) | 5 (18%) | 13 (35%) |  |  |  |  |
| Yes | 67 (52%) | 20 (31%) | 23 (82%) | 24 (65%) |  |  |  |  |
| **Anticoagulant and Antiplatelet Medications** ^4,5^ |  |  |  |  | **<0.001*** | **<0.001*** | **<0.001*** | 0.28 |
| No | 66 (51%) | 58 (89%) | 5 (18%) | 3 (8%) |  |  |  |  |
| Yes | 64 (49%) | 7 (11%) | 23 (82%) | 34 (92%) |  |  |  |  |
| **Lipid Lowering Medications** ^4,5^ |  |  |  |  | **<0.001*** | **<0.001*** | **<0.001*** | 0.58 |
| No | 65 (50%) | 47 (72%) | 9 (32%) | 9 (24%) |  |  |  |  |
| Yes | 65 (50%) | 18 (28%) | 19 (68%) | 28 (76%) |  |  |  |  |
| **Total Cholesterol** ^2,3^ | 177.4 ± 47.9 | 200.4 ± 41.3 | 158.3 ± 48.3 | 151.9 ± 39.2 | **<0.001*** | **<0.001*** | **<0.001*** | 1.00 |
| **LDL** ^2,3^ | 96.0 ± 36.1 | 111.2 ± 31.7 | 84.5 ± 38.0 | 78.3 ± 31.0 | **<0.001*** | **0.01*** | **<0.001*** | 0.97 |
| **HDL** ^2,3^ | 61.8 ± 21.9 | 71.5 ± 21.7 | 51.4 ± 18.8 | 53.0 ± 16.9 | **<0.001*** | **<0.001*** | **<0.001*** | 1.00 |
| **Triglycerides** ^2,3^ | 99.3 ± 55.0 | 88.8 ± 53.5 | 112.5 ± 57.9 | 107.4 ± 53.2 | **0.008*** | **0.04*** | **0.03*** | 1.00 |
| **BMI** ^2,3^ | 27.4 ± 8.3 | 25.5 ± 7.6 | 29.6 ± 9.6 | 28.6 ± 7.9 | **0.02*** | 0.08 | 0.07 | 1.00 |
| ^1^ Mean ± (SD); n (%)  ^2^ Dunn’s test with Bonferroni Correction  ^3^ Kruskal-Wallis test  ^4^ Fisher’s exact test  ^5^ Pearson’s Chi-squared test | | | | | | | | |
