# Supplementary material for: Retinal ischemic perivascular lesions in cortical cerebral infarction: a comparison against controls
Source: Eye (Lond). 2026 Jun 27;40(13):2100–6. doi: 10.1038/s41433-026-04680-1 (PMC13538709; doi:10.1038/s41433-026-04680-1)
Supplement: Supplementary file 4 [file 41433_2026_4680_MOESM4_ESM.docx]

**Supplementary Table 2:** Demographic and clinical characteristics comparison between the anterior and posterior circulation groups

| **Characteristic** | **Post-Stroke**  **N = 37** ^1^ | **Anterior**  **N = 17** ^1^ | **Posterior**  **N = 20** ^1^ | **P-value** |
| --- | --- | --- | --- | --- |
| **Age** ^2^ | 61.9 ± 15.9 | 61.0 ± 17.3 | 63.0 ± 15.0 | 0.68 |
| **Sex** ^3^ |  |  |  | 0.52 |
| Male | 20 (54%) | 8 (47%) | 12 (60%) |  |
| Female | 17 (46%) | 9 (53%) | 8 (40%) |  |
| **Hypertension** ^3^ |  |  |  | 0.32 |
| No | 20 (54%) | 11 (65%) | 9 (45%) |  |
| Yes | 17 (46%) | 6 (35%) | 11 (55%) |  |
| **Type 2 Diabetes Mellitus** ^3^ |  |  |  | 0.10 |
| No | 30 (81%) | 16 (94%) | 14 (70%) |  |
| Yes | 7 (19%) | 1 (6%) | 6 (30%) |  |
| **HbA1c** ^2^ | 5.66 ± 0.61 | 5.67 ± 0.54 | 5.65 ± 0.69 | 1.00 |
|  |  |  |  |  |
| **Smoking Status** ^4^ |  |  |  | 0.27 |
| Never | 24 (65%) | 9 (53%) | 15 (75%) |  |
| Former | 12 (32%) | 7 (41%) | 5 (25%) |  |
| Current | 1 (3%) | 1 (6%) | 0 (0%) |  |
| **Chronic Kidney Disease** ^3^ |  |  |  | 1.00 |
| No | 27 (73%) | 12 (71%) | 15 (75%) |  |
| Yes | 10 (27%) | 5 (29%) | 5 (25%) |  |
| **Peripheral Artery Disease** ^3^ |  |  |  | 1.00 |
| No | 37 (100%) | 17 (100%) | 20 (100%) |  |
| Yes | 0 (0%) | 0 (0%) | 0 (0%) |  |
| **Atrial Fibrillation** ^3^ |  |  |  | 0.68 |
| No | 30 (81%) | 13 (76%) | 17 (85%) |  |
| Yes | 7 (19%) | 4 (24%) | 3 (15%) |  |
| **Carotid Artery Stenosis** ^3^ |  |  |  | 0.28 |
| No | 26 (70%) | 10 (59%) | 16 (80%) |  |
| Yes | 11 (30%) | 7 (41%) | 4 (20%) |  |
| **History of CVD** ^3^ |  |  |  | 1.00 |
| No | 12 (44%) | 5 (29%) | 7 (35%) |  |
| Yes | 25 (56%) | 12 (71%) | 13 (65%) |  |
| **Anti-hypertensive Medications** ^3^ |  |  |  | 0.73 |
| No | 13 (35%) | 5 (29%) | 8 (40%) |  |
| Yes | 24 (65%) | 12 (71%) | 12 (60%) |  |
| **Anticoagulant and Antiplatelet Medications** ^3^ |  |  |  | 0.09 |
| No | 3 (8%) | 3 (18%) | 0 (0%) |  |
| Yes | 34 (92%) | 14 (82%) | 20 (100%) |  |
| **Lipid Lowering Medications** ^3^ |  |  |  | 0.25 |
| No | 9 (24%) | 6 (35%) | 3 (15%) |  |
| Yes | 28 (76%) | 11 (65%) | 17 (85%) |  |
| **Total Cholesterol** ^2^ | 151.9 ± 39.2 | 146.5 ± 43.6 | 156.6 ± 35.6 | 0.38 |
| **LDL** ^2^ | 78.3 ± 31.0 | 73.1 ± 33.4 | 82.7 ± 29.0 | 0.29 |
| **HDL** ^2^ | 53.0 ± 16.9 | 51.3 ± 18.8 | 54.5 ± 15.4 | 0.54 |
| **Triglycerides** ^2^ | 107.4 ± 53.2 | 115.6 ± 66.0 | 100.5 ± 39.7 | 0.68 |
| **BMI** ^2^ | 28.6 ± 7.9 | 27.3 ± 4.5 | 29.7 ± 9.94 | 0.80 |
| ^1^ Mean ± (SD); n (%)  ^2^ Wilcox rank sum test  ^3^ Fisher’s exact test  ^4^ Pearson’s Chi-squared test | | | | |
