# Supplementary material for: Retinal ischemic perivascular lesions in cortical cerebral infarction: a comparison against controls
Source: Eye (Lond). 2026 Jun 27;40(13):2100–6. doi: 10.1038/s41433-026-04680-1 (PMC13538709; doi:10.1038/s41433-026-04680-1)
Supplement: Supplementary file 5 [file 41433_2026_4680_MOESM5_ESM.docx]

**Supplementary Table 3:** Comparison of retinal ischemic perivascular lesions between the anterior and posterior circulation sub-groups derived from the post-stroke group

| **Characteristic** | **Post-Stroke**  **N = 37**^1^ | **Anterior**  **N = 17**^1^ | **Posterior**  **N = 20**^1^ | **P-value** |
| --- | --- | --- | --- | --- |
| **RIPL Score** ^2^ | 3.62 ± 4.14 | 3.82 ± 5.56 | 3.45 ± 2.52 | 0.29 |
| **Categorized RIPL score**^3^ |  |  |  | 1.00 |
| 0-1 | 11 (30%) | 5 (29%) | 6 (30%) |  |
| 2+ | 26 (70%) | 12 (71%) | 14 (70%) |  |
| ^1^ Mean ± (SD); n (%)  ^2^ Wilcox rank sum test  ^3^ Fisher’s exact test | | | | |
