# Supplementary material for: Retinal ischemic perivascular lesions in cortical cerebral infarction: a comparison against controls
Source: Eye (Lond). 2026 Jun 27;40(13):2100–6. doi: 10.1038/s41433-026-04680-1 (PMC13538709; doi:10.1038/s41433-026-04680-1)
Supplement: Supplementary file 6 [file 41433_2026_4680_MOESM6_ESM.docx]

**Supplementary Table 4:** Comparison of retinal ischemic perivascular lesions between ipsilateral and contralateral stroke hemispheres derived from the post-stroke group

| **Characteristic** | **Post-Unilateral Stroke Eyes**  **N = 68**^1^ | **Ipsilateral Eye**  **N = 34**^1^ | **Contralateral Eye**  **N = 34**^1^ | **P-value** |
| --- | --- | --- | --- | --- |
| **RIPL Score** ^2^ | 1.82 ± 2.68 | 2.03 ± 2.79 | 1.62 ± 2.59 | 0.12 |
| **Categorized RIPL score**^3^ |  |  |  | 0.29 |
| 0-1 | 39 (57%) | 18 (53%) | 621 (62%) |  |
| 2+ | 29 (43%) | 16 (47%) | 13 (38%) |  |
| ^1^ Mean ± (SD); n (%)  ^2^ Wilcox rank sum test  ^3^ Fisher’s exact test | | | | |
