# Supplementary material for: Retinal ischemic perivascular lesions in cortical cerebral infarction: a comparison against controls
Source: Eye (Lond). 2026 Jun 27;40(13):2100–6. doi: 10.1038/s41433-026-04680-1 (PMC13538709; doi:10.1038/s41433-026-04680-1)
Supplement: Supplementary file 7 [file 41433_2026_4680_MOESM7_ESM.docx]

**Supplementary Table 5:** Retinal ischemic perivascular lesions (RIPL) counts per participant and per eye

| **Participant Number** | **Stroke Status** | **Total RIPL** | **RIPL OD** | **RIPL OS** |
| --- | --- | --- | --- | --- |
| Participant 1 | Control | 1 | 0 | 1 |
| Participant 2 | Control | 1 | 0 | 1 |
| Participant 3 | Control | 1 | 1 | 0 |
| Participant 4 | Control | 2 | 2 | 0 |
| Participant 5 | Control | 0 | 0 | 0 |
| Participant 6 | Control | 2 | 2 | 0 |
| Participant 7 | Control | 3 | 2 | 1 |
| Participant 8 | Control | 4 | 0 | 4 |
| Participant 9 | Control | 3 | 1 | 2 |
| Participant 10 | Control | 2 | 1 | 1 |
| Participant 11 | Control | 0 | 0 | 0 |
| Participant 12 | Control | 0 | 0 | 0 |
| Participant 13 | Control | 4 | 2 | 2 |
| Participant 14 | Control | 4 | 2 | 2 |
| Participant 15 | Control | 14 | 12 | 2 |
| Participant 16 | Control | 3 | 2 | 1 |
| Participant 17 | Control | 3 | 2 | 1 |
| Participant 18 | Control | 7 | 5 | 2 |
| Participant 19 | Control | 2 | 1 | 1 |
| Participant 20 | Control | 6 | 2 | 4 |
| Participant 21 | Control | 4 | 3 | 1 |
| Participant 22 | Control | 2 | 0 | 2 |
| Participant 23 | Control | 1 | 1 | 0 |
| Participant 24 | Control | 0 | 0 | 0 |
| Participant 25 | Control | 3 | 2 | 1 |
| Participant 26 | Control | 0 | 0 | 0 |
| Participant 27 | Control | 3 | 2 | 1 |
| Participant 28 | Control | 4 | 3 | 1 |
| Participant 29 | Control | 0 | 0 | 0 |
| Participant 30 | Control | 1 | 0 | 1 |
| Participant 31 | Control | 4 | 4 | 0 |
| Participant 32 | Control | 0 | 0 | 0 |
| Participant 33 | Control | 0 | 0 | 0 |
| Participant 34 | Control | 0 | 0 | 0 |
| Participant 35 | Control | 0 | 0 | 0 |
| Participant 36 | Control | 1 | 1 | 0 |
| Participant 37 | Control | 4 | 1 | 3 |
| Participant 38 | Control | 2 | 1 | 1 |
| Participant 39 | Control | 1 | 1 | 0 |
| Participant 40 | Control | 1 | 0 | 1 |
| Participant 41 | Control | 2 | 0 | 2 |
| Participant 42 | Control | 2 | 2 | 0 |
| Participant 43 | Control | 1 | 1 | 0 |
| Participant 44 | Control | 1 | 0 | 1 |
| Participant 45 | Control | 1 | 0 | 1 |
| Participant 46 | Control | 1 | 0 | 1 |
| Participant 47 | Control | 2 | 1 | 1 |
| Participant 48 | Control | 7 | 4 | 3 |
| Participant 49 | Control | 0 | 0 | 0 |
| Participant 50 | Control | 0 | 0 | 0 |
| Participant 51 | Control | 1 | 1 | 0 |
| Participant 52 | Control | 3 | 2 | 1 |
| Participant 53 | Control | 1 | 0 | 1 |
| Participant 54 | Control | 3 | 1 | 2 |
| Participant 55 | Control | 0 | 0 | 0 |
| Participant 56 | Control | 2 | 0 | 2 |
| Participant 57 | Control | 0 | 0 | 0 |
| Participant 58 | Control | 0 | 0 | 0 |
| Participant 59 | Control | 0 | 0 | 0 |
| Participant 60 | Control | 7 | 3 | 4 |
| Participant 61 | Control | 0 | 0 | 0 |
| Participant 62 | Control | 0 | 0 | 0 |
| Participant 63 | Control | 1 | 0 | 1 |
| Participant 64 | Control | 0 | 0 | 0 |
| Participant 65 | Control | 5 | 2 | 3 |
| Participant 66 | Post-Stroke | 1 | 1 | 0 |
| Participant 67 | Post-Stroke | 4 | 2 | 2 |
| Participant 68 | Post-Stroke | 3 | 3 | 0 |
| Participant 69 | Post-Stroke | 2 | 1 | 1 |
| Participant 70 | Post-Stroke | 15 | 14 | 1 |
| Participant 71 | Post-Stroke | 4 | 4 | 0 |
| Participant 72 | Post-Stroke | 4 | 4 | 0 |
| Participant 73 | Post-Stroke | 1 | 0 | 1 |
| Participant 74 | Post-Stroke | 1 | 0 | 1 |
| Participant 75 | Post-Stroke | 3 | 2 | 1 |
| Participant 76 | Post-Stroke | 2 | 0 | 2 |
| Participant 77 | Post-Stroke | 21 | 15 | 6 |
| Participant 78 | Post-Stroke | 1 | 0 | 1 |
| Participant 79 | Post-Stroke | 4 | 1 | 3 |
| Participant 80 | Post-Stroke | 4 | 3 | 1 |
| Participant 81 | Post-Stroke | 0 | 0 | 0 |
| Participant 82 | Post-Stroke | 0 | 0 | 0 |
| Participant 83 | Post-Stroke | 6 | 4 | 2 |
| Participant 84 | Post-Stroke | 4 | 2 | 2 |
| Participant 85 | Post-Stroke | 1 | 0 | 1 |
| Participant 86 | Post-Stroke | 2 | 1 | 1 |
| Participant 87 | Post-Stroke | 9 | 3 | 6 |
| Participant 88 | Post-Stroke | 4 | 2 | 2 |
| Participant 89 | Post-Stroke | 2 | 0 | 2 |
| Participant 90 | Post-Stroke | 2 | 2 | 0 |
| Participant 91 | Post-Stroke | 6 | 3 | 3 |
| Participant 92 | Post-Stroke | 4 | 0 | 4 |
| Participant 93 | Post-Stroke | 6 | 3 | 3 |
| Participant 94 | Post-Stroke | 7 | 2 | 5 |
| Participant 95 | Post-Stroke | 2 | 1 | 1 |
| Participant 96 | Post-Stroke | 3 | 1 | 2 |
| Participant 97 | Post-Stroke | 3 | 2 | 1 |
| Participant 98 | Post-Stroke | 1 | 0 | 1 |
| Participant 99 | Post-Stroke | 0 | 0 | 0 |
| Participant 100 | Post-Stroke | 2 | 1 | 1 |
| Participant 101 | Post-Stroke | 0 | 0 | 0 |
| Participant 102 | Post-Stroke | 0 | 0 | 0 |
| Participant 67 | Pre-Stroke | 2 | 1 | 1 |
| Participant 79 | Pre-Stroke | 2 | 0 | 2 |
| Participant 95 | Pre-Stroke | 3 | 2 | 1 |
| Participant 98 | Pre-Stroke | 0 | 0 | 0 |
| Participant 102 | Pre-Stroke | 9 | 4 | 5 |
| Participant 103 | Pre-Stroke | 5 | 2 | 3 |
| Participant 104 | Pre-Stroke | 11 | 4 | 7 |
| Participant 105 | Pre-Stroke | 2 | 1 | 1 |
| Participant 106 | Pre-Stroke | 1 | 0 | 1 |
| Participant 107 | Pre-Stroke | 3 | 1 | 2 |
| Participant 108 | Pre-Stroke | 1 | 1 | 0 |
| Participant 109 | Pre-Stroke | 0 | 0 | 0 |
| Participant 110 | Pre-Stroke | 0 | 0 | 0 |
| Participant 111 | Pre-Stroke | 4 | 1 | 3 |
| Participant 112 | Pre-Stroke | 1 | 0 | 1 |
| Participant 113 | Pre-Stroke | 6 | 4 | 2 |
| Participant 114 | Pre-Stroke | 13 | 7 | 6 |
| Participant 115 | Pre-Stroke | 3 | 0 | 3 |
| Participant 116 | Pre-Stroke | 0 | 0 | 0 |
| Participant 117 | Pre-Stroke | 3 | 1 | 2 |
| Participant 118 | Pre-Stroke | 1 | 1 | 0 |
| Participant 119 | Pre-Stroke | 0 | 0 | 0 |
| Participant 120 | Pre-Stroke | 9 | 7 | 2 |
| Participant 121 | Pre-Stroke | 7 | 5 | 2 |
| Participant 122 | Pre-Stroke | 5 | 2 | 3 |
| Participant 123 | Pre-Stroke | 2 | 0 | 2 |
| Participant 124 | Pre-Stroke | 0 | 0 | 0 |
