# Supplementary material for: Retinal ischemic perivascular lesions in cortical cerebral infarction: a comparison against controls
Source: Eye (Lond). 2026 Jun 27;40(13):2100–6. doi: 10.1038/s41433-026-04680-1 (PMC13538709; doi:10.1038/s41433-026-04680-1)
Supplement: Supplementary file 8 [file 41433_2026_4680_MOESM8_ESM.docx]

**Supplementary Table 6:** Univariate logistic regression models assessing the relationship between elevated retinal ischemic perivascular lesions (RIPL) presence (RIPL ≥ 2) and decreased RIPL (RIPL < 2) presence between the control and post-stroke group.

| **Population** | **Odds Ratio** | **95% Confidence Interval** | **Beta Coefficient** | **P-value** |
| --- | --- | --- | --- | --- |
| Control | - | - |  |  |
| Pre-Stroke | 1.77 | 0.79-4.92 | -0.680 | 0.14 |
| Control | - | - |  |  |
| Post-Stroke | 2.59 | 1.10-6.11 | 0.9526 | **0.03*** |
| Pre-Stroke | - | - |  |  |
| Post-Stroke | 1.31 | 0.46-3.74 | 0.272 | 0.61 |
